# Autophagy Plays a Suppressive Role in Bladder Tumor Formation in an Orthotopic Mouse Model and Bladder Cancer Patient Specimens

**DOI:** 10.1002/kjm2.70179

**Published:** 2026-01-23

**Authors:** Wan‐Ting Kuo, Chin‐Chen Pan, Yi‐Wen Liu, Nan‐Haw Chow, Hong‐Lin Cheng, Shan‐Ying Wu, Sheng‐Hui Lan, Chih‐Peng Chang, Hsiao‐Sheng Liu

**Affiliations:** ^1^ Graduate Institute of Integrated Medicine China Medical University Taichung Taiwan; ^2^ Department of Microbiology and Immunology, College of Medicine National Cheng Kung University Tainan Taiwan; ^3^ Department of Pathology and Laboratory Medicine Taipei Veterans General Hospital Taipei Taiwan; ^4^ Department of Microbiology, Immunology and Biopharmaceuticals National Chiayi University Chiayi Taiwan; ^5^ Department of Pathology National Cheng Kung University, College of Medicine Tainan Taiwan; ^6^ Center for Precision Medicine China Medical University Hospital, China Medical University Taichung Taiwan; ^7^ Department of Urology College of Medicine, National Cheng Kung University Tainan Taiwan; ^8^ Department of Microbiology and Immunology, College of Medicine Taipei Medical University Taipei Taiwan; ^9^ Graduate Institute of Medical Sciences, College of Medicine Taipei Medical University Taipei Taiwan; ^10^ Department of Life Sciences and Institute of Genome Sciences National Yang Ming Chiao Tung University Taipei Taiwan; ^11^ Cancer and Immunology Research Center National Yang Ming Chiao Tung University Taipei Taiwan; ^12^ M. Sc. Program in Tropical Medicine, College of Medicine Kaohsiung Medical University Kaohsiung Taiwan; ^13^ Center for Cancer Research, College of Medicine Kaohsiung Medical University Kaohsiung Taiwan

**Keywords:** amiodarone, autophagy, bladder cancer, intravesical instillation, orthotopic tumor model

## Abstract

Autophagy plays either a suppressing or promoting role during tumor development. Clarifying the role of autophagy in bladder tumorigenesis both in vitro and in vivo is crucial for developing novel therapeutic strategies through manipulating autophagy activity. Herein, we noninvasively monitored how autophagy affects bladder tumor formation using a murine model of orthotopic bladder tumor formation by “in vivo imaging system (IVIS) and transabdominal micro‐ultrasound imaging (MUI)” and validated the notion in cell lines and bladder cancer patients. Mimic clinical administration, all the drugs were delivered into the urinary bladder of the mice via intravesical instillation. Plasma biochemistry parameters, hemograms, blood pressure, and blood concentration of amiodarone and desethylamiodarone were analyzed in treated mice. Low autophagy activity was detected in the bladder tumors and associated with poor overall survival of bladder cancer patients. Amiodarone‐induced autophagy activity suppressed bladder tumor formation, whereas silencing Atg5 expression reversed the suppression. Notably, amiodarone showed equivalent anti‐tumor efficacy but with fewer side effects on the treated mice compared to the clinical anti‐cancer drug Mitomycin C (MMC). Furthermore, amiodarone delivered by intravesical route showed a negligible influence on the physiologic conditions of the treated mice. Our orthotopic mouse model revealed that increasing autophagy activity alleviated bladder tumor development. Similarly, low autophagy is associated with a poor overall survival rate. Furthermore, repurposing amiodarone‐induced autophagy accompanied by trivial side effects shows potential for the treatment of bladder cancer patients.

**Abbreviations**
BC: bladder cancer
BUN: blood urea nitrogen
CQ: chloroquine
GOT: glutamate oxaloacetate transaminase
GPT: glutamate pyruvate transaminase
Hb: hemoglobin concentration
HPLC‐UV: high performance liquid chromatography‐ultraviolet
IVIS: in vivo imaging system
Ly: lymphocyte
MMC: mitomycin C
MUI: micro‐ultrasound image
PLT: platelets
RBC: red blood cell
WBC: white blood cell

## 1 Introduction

Bladder cancer (BC) is among the top 10 most common cancers worldwide and occurs more frequently in men than in women [1]. Transitional cell carcinoma originating from transitional epithelial cells of the urinary tract accounts for nearly 95% of BC, and the remaining 5% is squamous cell carcinoma. In terms of clinical treatment, BC patients are managed by transurethral resection, followed by intravesical administration of therapeutic agents. After either intravesical immunotherapy or chemotherapy, approximately 80% of patients with superficial bladder cancers relapse, and 10% of them progress to muscle‐invasive carcinoma [2]. Most intravesical therapeutic agents, including Bacillus Calmette‐Guérin, Mitomycin C (MMC), and doxorubicin, result in chemical cystitis and decreased bladder capacity [3]. Therefore, there is an urgent demand for new therapeutic agents to improve treatment.

Autophagy is a catabolic process by which the phagophore is initially formed followed by the membrane elongation and the formation of the autophagosome, which recruits damaged or unnecessary organelles and materials into the autophagosome and then fuses with the lysosome for recycling. The entire process is termed autophagic flux. Dysfunctional autophagy leads to various diseases including cancers. Autophagy plays controversial roles in diverse cancers depending on the tumor type and stage [4]. Autophagy suppressing or promoting bladder tumor formation has been reported [5, 6, 7]. Therefore, it remains contradictory on the role of autophagy in the tumorigenesis and therapy of bladder cancer. It is difficult to clarify this discrepancy due to the lack of optimal animal models and sensitive noninvasive tools to spatiotemporally monitor bladder tumor formation in vivo. Amiodarone is a widely used antiarrhythmic drug in clinical practice and blocks calcium channels to induce autophagic activity [8]. We previously reported that amiodarone acts as a repurposed drug that facilitates autophagic activity through the selective degradation of the oncogenic factors miR‐224 and cyclin D1, thereby suppressing liver cancer progression [9, 10]. Thus, amiodarone may represent a potential anti‐cancer agent for the treatment of bladder cancer.

Intravesical therapy is widely used to extend the recurrence‐free interval of bladder cancer patients after transurethral resection of the bladder tumor [11]. However, using this therapy, it is not possible to treat the early phase of bladder tumor formation without a reliable noninvasive monitoring tool. The current in vivo imaging instruments to monitor murine bladder cancer development include magnetic resonance imaging, bioluminescent or fluorescent imaging, and intravesical ultrasound. Among them, bioluminescent imaging is widely used to monitor tumor growth by detecting the bioluminescence of cancer cells harboring the luciferase reporter gene [12, 13]. Micro‐ultrasound imaging (MUI) provides two‐ and three‐dimensional images of tumors, thereby allowing noninvasive observation of tumor growth and evaluation of tumor volume. Based on the above knowledge, the application of combined imaging systems of bioluminescence and transabdominal MUI becomes the most sensitive and reliable approach to investigate the early phase of bladder tumor formation in a spatiotemporal fashion in an orthotopic bladder tumor model [14, 15], by which the tumor appearance can be detected with high accuracy before initiation of treatment. Moreover, tumor growth or regression can be followed up in vivo longitudinally.

This study aimed to clarify the effect of increased autophagy on bladder cancer tumorigenesis both in vitro and in an orthotopic mouse model of bladder tumor formation, and to validate the clinical significance of autophagy in bladder cancer patient specimens. Moreover, we demonstrated that intravesical instillation of amiodarone as a potential repurposing drug effectively suppressed bladder tumor formation in an orthotopic mouse tumor model with trivial side effects.

## 2 Materials and Methods

### 2.1 Cell Lines and Culture

The carcinogen‐induced transitional mouse cell carcinoma cell line MB49 was derived from C57BL/6 mice (a gift from Dr. Yi‐Wen Liu, National Chiayi University, Chiayi, Taiwan) [16]. MB49 cells were cultured in Roswell Park Memorial Institute 1640 medium (RPMI) (GIBCO, MD, USA) supplemented with 10% fetal bovine serum (FBS) and 1% penicillin–streptomycin. MB49 transfected with luciferase gene (MB49‐Luc) cell lines were maintained under the same conditions in the presence of 400 μg/mL of G418 (Merck, Darmstadt, Germany). MB49LucshAtg5 and MB49LucshLacZ cell lines were cultured in RPMI with 2 μg/mL of puromycin and 10% FBS. All cell lines were incubated at 37°C in a 5% CO_2_ incubator.

### 2.2 Flow Cytometry Assay

To determine the induction of apoptosis, cells were stained with Annexin V‐FITC/PI Apoptosis Detection Kit (Elabscience, China) before flow cytometry (BD FACSCalibur, RRID:SCR_000401).

### 2.3 Cytotoxicity Assay

Cytotoxicity of amiodarone on MB49LucshLacZ and MB49LucshAtg5 cells was determined by MTT (3‐(4,5‐Dimethylthiazol‐2‐yl)‐2,5‐Diphenyltetrazolium Bromide) assay (Sigma). The absorbance was measured at 540 nm wavelength using an ELISA Reader (Thermo Lab Systems, Franklin, MA).

### 2.4 Short Hairpin RNA (shRNA) Lentiviral System

We purchased lentiviruses with shRNA constructs targeting LacZ and Atg5 from National RNAi Core Facility (Academia Sinica, Taiwan). The ID numbers of the effective clones are as follows: TRCN0000072229 for clone shLacZ and TRCN0000099433 for clone shAtg5. MB49 cells were infected by lentivirus. Clone selection was conducted by 2 μg/mL puromycin treatment for 2 weeks.

### 2.5 Clinical Bladder Cancer Patient Specimens

The tissue array of human bladder cancer specimens (289 patients) was provided by Dr. Chin‐Chen Pan, Taipei Veterans General Hospital, Taipei, Taiwan. Informed consent was obtained from all patients, and the Institutional Review Board of Taipei Veterans General Hospital approved this study (IRB document number: VGHIRB 2012‐03‐009A).

### 2.6 Orthotopic Mouse Model of Bladder Tumor

Female C57BL/6 mice (5–6 weeks old) were obtained from the Laboratory Animal Center of National Cheng Kung University (Tainan, Taiwan). The experimental protocol complied with Taiwan's Animal Protection Act and was approved by the Laboratory Animal Care and Use Committee of National Cheng Kung University (document number: IACUC103023). Mice were anesthetized by intraperitoneal administration of Zoletil 50 solution (10 mg/mL, Virbac, Taipei, Taiwan) combined with 0.5 mL of Rompun solution (Bayer, Germany) and 19.5 mL of normal saline at a dose of 0.08 to 0.1 mL/10 g of body weight. Subsequently, the soft‐tipped end of a 24‐gauge teflon intravenous catheter (BD, NJ, USA) was gently inserted into the bladder via the urethra until it reached the bladder wall. A chemical lesion was generated on the inner wall of the bladder by intravesical instillation of 50 μL of AgNO_3_ (0.1 M). An adequate and controlled diffuse cauterization of the bladder wall was produced. The injected solution was washed out by transurethral infusion of 300 μL of PBS after 10 s. The suspension of mouse bladder cancer MB49Luc cells (1 × 10^7^) was then injected and preserved in the bladder for 1 h. One group of mice (*N* = 7) was intravesically inoculated with 1.5 mg/50 μL amiodarone (Sigma) dissolved in PBS and 40% DMSO. Another group (*N* = 7) was intravesically inoculated with 100 μg/50 μL MMC (Kyowa, Tokyo, Japan) dissolved in PBS. The control group of mice (*N* = 7) was intravesically treated with PBS and 40% DMSO. The intravesical injection of amiodarone or MMC was performed from day 4 and repeated at 3‐day intervals until day 19 after cancer cell inoculation. All of the mice were sacrificed on day 21, and the bladder volumes and weights were measured. Three independent experiments were performed in triplicate. Another drug experiment using shLacZ and shAtg5 cells in mice (*N* = 3/each group) started from day 7 and all mice were sacrificed on day 33. The bladder volumes and weights were measured.

### 2.7 Western Blotting

Protein was extracted from the cells after various treatments by treatment with the lysis buffer. Protein samples were separated using 12% Sodium dodecyl sulfate‐polyacrylamide gel and then transferred to a nitrocellulose membrane (Millipore, MA, USA) in transfer buffer (0.025 M Tris‐Base, 0.2 M glycine) at 100 V for 1.5 h using an electroblotter (Amersham Pharmacia Biosciences Corp, NJ, USA). Transferring membranes were rinsed with 5% nonfat milk for 1 h. Antibodies against β‐actin (1:5000; Mouse monoclonal, clone AC‐74, Sigma), LC3 (1:5000; Rabbit polyclonal, PM036, MBL International), Atg5 (1:1000; Rabbit monoclonal, Cell signaling), caspase 3, PARP1 (GeneTex, Taiwan), and p62 (MBL) were used to detect the specific proteins.

### 2.8 Immunohistochemical (IHC) Staining

Mice tissues were fixed with 4% formalin and embedded in paraffin. Paraffin sections were treated with anti‐Ki67 (1:100, Rabbit monoclonal, Clone SP6, Spring Bioscience), Atg5 (1:100, abcam, MA, USA), p62 (1:500, Rabbit polyclonal, PM045, MBL International), or LC3 antibody (1:5000; Rabbit polyclonal, PM036, MBL International) overnight at 4°C. The slides were labeled with a secondary antibody conjugated with horseradish peroxidase (Dako, CA, USA). AEC (Dako) as a substrate of HRP was used to react with the antibody recognition proteins. Nucleus was stained with hematoxylin (Merck). Whole slides were scanned at 20× magnification for visualization of the tumor; three non‐overlapping regions of interest (ROI) were selected for quantification. The size of the ROI was 90 × 90 μm (0.0081 mm^2^) in each mouse.

### 2.9 In Vivo Imaging System (IVIS)

In vitro assay*:* Different cell numbers were seeded into a 96‐well plate and then treated with 50 μL of D‐luciferin (15 mg/mL, Perkin Elmer, Massachusetts, USA). The luminescence was determined by IVIS (Caliper Life Sciences, MA, USA). In vivo assay*:* The luminescence in mice was measured by IVIS (Caliper Life Sciences) after intraperitoneal injection of 10 μL/g of body weight of D‐luciferin (15 mg/mL).

### 2.10 Immunofluorescent Staining

Paraffin‐embedded tissue sections on the slides were incubated for 30 min in 0.1% Triton X‐100 in PBS followed by treatment with anti‐LC3 antibody (1:5000; Rabbit polyclonal, PM036, MBL International) overnight at 4°C. The primary antibody was labeled using the specific secondary antibody with Alexa Fluor 568 (Invitrogen, A11004). The nucleus was stained with Hoechst (1:200) (5 mg/mL, Sigma) for 20 min. The dye on the slide was washed out by PBS for 1 h. The fluorescent image was observed under a multiphoton confocal microscope (Olympus, FV‐1000MPE, Tokyo, Japan).

### 2.11 High‐Performance Liquid Chromatography‐Ultraviolet (HPLC‐UV) Detection

HPLC‐UV detection and analysis in this study was performed using an Agilent‐1200 Infinity system (Agilent Technologies, Santa Clara, CA, USA). We used Pursuit 5 C18 100 × 3.0 mm column (Agilent Technologies) to analyze our samples and MetaGuard 4.6 mm Pursuit 5u C18 (Agilent Technologies, Santa Clara, CA, USA) was used to protect our column. A 250 μL pulled point conical glass insert was used to load our samples for analysis. A mobile phase of acetonitrile (sigma) and 0.01% diethylamine (sigma) in water (80/20, v/v) was performed for an isocratic run of 15 min. The flow rate was 1.0 mL/min, and the UV‐detector was set at a wavelength of 245 nm.

Stock solutions of amiodarone (sigma) and desethylamiodarone (sigma) of 1000 μg/mL were prepared in methanol and stored at −20°C. The stock solutions of amiodarone and desethylamiodarone were diluted with acetonitrile to serve as working solutions containing 0, 0.32, 0.8, 1.6, 2, 8, and 16 μg/mL amiodarone and desethylamiodarone. We then added 50 μL of the working solutions or 50 μL acetonitrile to 100 μL of plasma and then obtained amiodarone and desethylamiodarone concentrations of 0, 0.16, 0.4, 0.8, 1, 4, and 8 μg/mL in plasma. All of the working solutions were stored in a refrigerator (2°C to 8°C).

Acetonitrile was added to all of the samples to a final volume of 300 μL. After vortexing for 30 s, the samples were centrifuged at 10,000 × g for 10 min. The supernatants were transferred into individual Eppendorf tubes, and 50 μL of sample was injected into the HPLC column for analysis.

### 2.12 Blood Pressure Analysis

The BP‐2000 Blood Pressure Analysis System (Visitech Systems, Norway) was used to measure the tail pressure of mice, which represented the blood pressure of the mice. This system can measure multiple mice simultaneously and in all of the mice, measurements of the tail pressure were conducted at the end of treatment.

### 2.13 Transabdominal Micro‐Ultrasound Imaging (MUI)

The anesthetized C57BL/6 mice were secured to a heated ultrasound platform after the abdominal fur had been shaved. The mouse bladder was catheterized followed by injection of 100 μL of PBS to fill the bladder for better visualization under ultrasound monitoring. High viscosity ultrasound gel was applied over the abdomen of the mouse. A Vevo770 high‐frequency ultrasound system (Visual Sonics, Toronto, Canada) was used to view the internal image of the mouse bladder for assessment of tumor formation.

### 2.14 Statistical Analysis

All data are presented as the mean ± standard deviation. Comparisons between different groups were analyzed by Student's *t*‐test or one‐way ANOVA. The analysis of survival rate was performed by Kaplan–Meier survival analysis, which included the use of log‐rank (Mentel‐Cox). Software GraphPad Prism 5.0 was used for statistical analyses. *p*‐values were considered significant under the following conditions: *: *p* < 0 0.05; **: *p* < 0.01; ***: *p* < 0 0.001.

## 3 Results

### 3.1 Autophagy Activity Is Inversely Correlated With Bladder Cancer Stages and Overall Survival Rate of Bladder Cancer Patients

**FIGURE 1:**
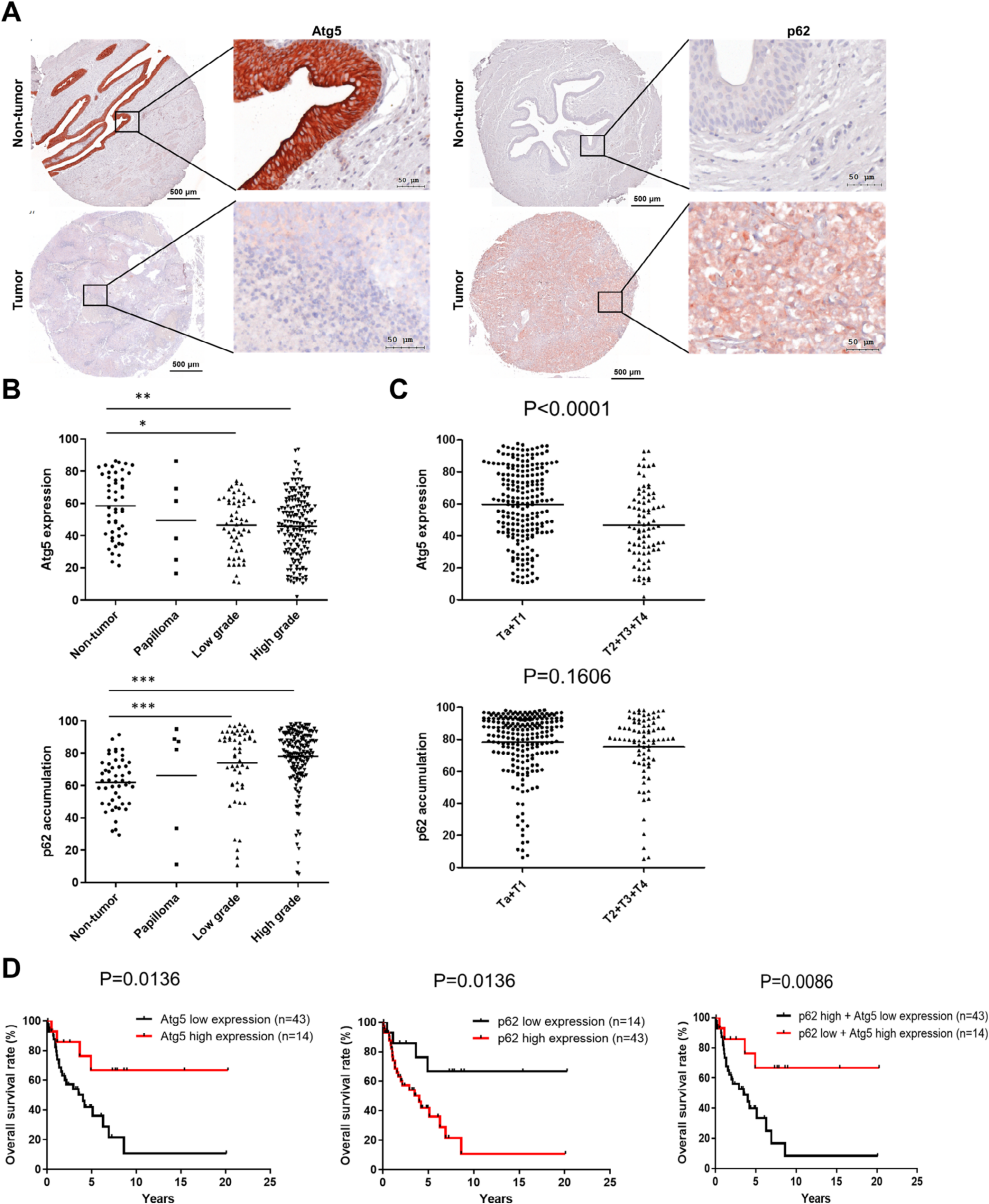
Autophagy activity is inversely correlated with bladder cancer stages and overall survival rate of bladder cancer patients. (A) The protein levels of Atg5 and p62 in the tumor and non‐tumor parts of 289 bladder cancer specimens were investigated by IHC staining. (B, C) The protein levels of Atg5 and p62 in different bladder tumor grades and different bladder tumor stages were measured by IHC staining and were quantified by HistoQuest analysis software. The percentage of Atg5 and p62 expression in bladder tissues was calculated by cells with protein expression vs. all nucleated cells. (D) Overall survival rate of 57 patients with bladder cancer based on p62 and Atg5 protein levels. The overall survival rate was generated by Kaplan–Meier survival analysis followed by log rank test (Mentel‐Cox) to obtain the *p*‐value. *p < 0.05; **p < 0.01; ***p < 0.001. Data were analyzed by one‐way ANOVA.

The role of autophagy in bladder cancer tumorigenesis remains unclear; some studies have shown autophagy may promote bladder cancer, while others have found a mitigating effect on the development of bladder cancer. Therefore, we clarified the status of autophagy activities in the clinical specimens of bladder cancer patients in Taiwan. Autophagy activity in clinical cancer specimens has been evaluated by various markers representing autophagy activity including Atg5, Atg7, Beclin‐1, p62, and LC3 [17]. High Atg5 level represents increased autophagy activity. On the other hand, phosphorylated p62 at Ser403 and Ser349 has enhanced binding ability to ubiquitinated proteins for degradation [18]. Therefore, blocking autophagic degradation by bafilomycin A1 in human 5637 bladder cancer cells led to p62 accumulation, and further overexpressing or silencing the total form of p62 in bladder cancer cell lines can regulate cancer growth [19], suggesting that the total form of p62 accumulation represents impaired autophagic flux. In our studies, Atg5 expression and p62 accumulation levels were evaluated in 289 bladder cancer patient specimens in Taiwan. One representative bladder cancer patient specimen showed low Atg5 expression and high p62 accumulation in the tumor parts compared to the non‐tumor parts (Figure 1A). Based on categories of urothelial carcinoma, papilloma is a benign tumor that develops from the lining of the bladder. High‐grade tumors, on the other hand, grow rapidly and are more likely to metastasize. Our patient specimen analysis reveals the levels of Atg5 expression were significantly decreased in both low‐grade and high‐grade bladder cancer tumors compared to the adjacent normal parts (Figure 1B). In contrast, there was a significantly increased p62 accumulation in both low‐grade and high‐grade bladder tumors compared to the non‐tumor adjacent tissues (Figure 1B). We further classified the T stage bladder cancer specimens into non‐muscle invasive (Ta + T1) and muscle invasive (T2 + T3 + T4) groups. The level of Atg5 expression was significantly lower in the T2 + T3 + T4 group compared to the Ta + T1 group (Figure 1C). However, the level of p62 accumulation showed no significant difference between these two groups (Figure 1C). Furthermore, we analyzed 57 out of 289 bladder specimens with traceable records for survival data. Either low Atg5 expression or high p62 accumulation or low Atg5 + high p62 accumulation significantly correlated with the overall poor survival rate of bladder cancer patients in 57 specimens (*p* = 0.0136, p = 0.0136, *p* = 0.0086, Figure 1D). In summary, low autophagy activity correlates with high bladder cancer tumor grade and stage as well as poor overall survival rate of bladder cancer patients.

### 3.2 Evaluating the Anti‐Cancer Effects of Amiodarone and MMC on Bladder Tumor Formation of the Treated Mice

**FIGURE 2:**
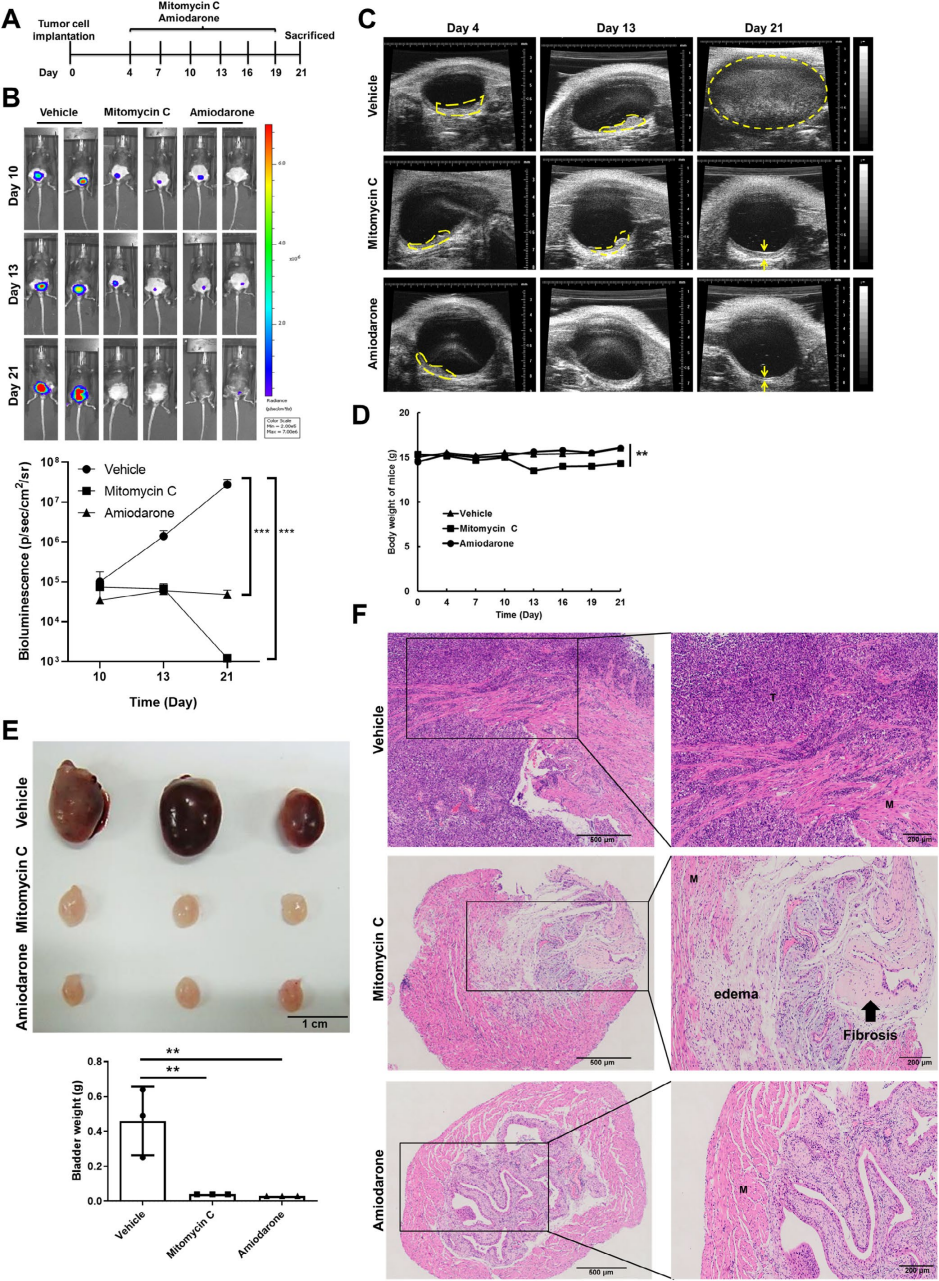
Evaluation of the anti‐cancer effects of amiodarone and MMC on bladder tumor formation of the treated mice. (A) The time course of the mice experiments. Four days after implantation of MB49Luc8 cells (1 × 10^7^) into the mice, amiodarone (1.5 mg/50 μL) or MMC (100 μg/50 μL) was intravesically instilled into the bladders. Vehicle represents PBS (1×) treatment. Each group contained seven mice. (B) Monitoring bioluminescence of the mice on days 10, 13, and 21 p.i. under IVIS. (C) Monitoring tumor formation in mice bladders after intravesical instillation on days 4, 13, and 21 p.i. under MUI system. Yellow arrow points to the region of edema in the bladder. (D) Measurements of the body weight of the mice from day 0 to day 21. (E) Morphology and quantification of the weight of the bladders after the mice were sacrificed on day 21 p.i. (F) H&E staining of the sections of bladder tissues of the mice. M, muscle; T, tumor. Error bars represent mean ± SD. *p < 0.05; **p < 0.01; ***p < 0.001. Data were analyzed by one‐way ANOVA.

To determine the role of autophagy in bladder cancer development in the orthotopic mouse model, we generated derivatives of mouse MB49 bladder cancer cells stably expressing luciferase reporter gene (MB49‐Luc). MB49Luc8 cells showing strong luminescence and high tumorigenicity both in vitro and in vivo were used in this study (Figure S1A–D). Additionally, tumor formation in mice bladders was spatiotemporally monitored in real time by two noninvasive instruments, MUI and IVIS. Small nodules in the bladder can be detected on day 4 p.i. using MUI (Figure S1E). Tumor formation on days 4, 13, and 21 p.i. was confirmed by H&E staining of sectioned bladder tissues (Figure S1E). We also used the MUI system to monitor the bladder tumor formation. In contrast, bioluminescent spots could not be detected in the implanted mice on day 4 p.i. by IVIS due to small tumor volume with weak bioluminescence; however, they become detectable on day 13 p.i. (Figure S1E). Our findings imply that MUI was more sensitive to detect early bladder tumor formation compared to IVIS, and both systems are reliable for noninvasively monitoring tumor formation and for clarifying the effect of autophagy on bladder tumor formation. MMC (Mitomycin C) is a chemotherapeutic agent with intravesical cytotoxicity against various cancers, including superficial bladder tumors, through the inhibition of DNA synthesis [17]. We previously reported that amiodarone, as an inducer of autophagy, significantly suppresses liver tumor formation [9, 10]. Herein, we used amiodarone to increase autophagy activity and used MMC as a positive control to compare the anti‐tumorigenesis and cytotoxicity of these two drugs in the orthotopic mouse model of bladder tumor formation. Briefly, we implanted the MB49Luc8 cells into the bladders of female C57BL/6 mice (5–6 weeks old) followed by intravesical inoculation of either amiodarone or MMC at 3‐day intervals from day 4 to day 19 post‐implantation (p.i.). All of the mice were sacrificed on day 21 p.i. (Figure 2A). We detected obvious bioluminescent spots at the external positions of the mice bladders from all groups on day 10 p.i. under IVIS. The sizes of the bioluminescent spots of the mice without drug treatment were larger on day 13 and day 21 p.i. (Vehicle, Figure 2B). In contrast, the bioluminescent spots of the groups treated with either amiodarone or MMC were smaller on day 10 p.i. and became undetectable compared to the vehicle group on day 13 and day 21 p.i. (Figure 2B). We further validated the results of IVIS using the MUI system. In contrast to the abovementioned results, we detected small nodules (yellow dash line, indicating tumor formation) on the internal smooth epithelial tissue of the bladders in all of the groups on day 4 p.i. under MUI. Amiodarone or MMC was then intravesically instilled into the mice bladders. Tumor sizes in the group without drug treatment were gradually increased from day 13 to day 21 p.i. (Vehicle, Figure 2C). In contrast, bladder tumor formation was greatly suppressed in the groups treated with either amiodarone or MMC on day 13 and day 21 p.i. (Figure 2C). However, we found that in MMC‐treated mice, the epithelial layer of the internal bladder tissue had become thicker on day 21 p.i. compared to the amiodarone‐treated mice (the third column, yellow arrow, Figure 2C). The body weights of the mice were measured over a period of 21 days. We found that only the MMC‐treated mice showed significantly decreased body weight from day 13 to day 21 p.i., and the other two groups showed no difference in body weight (Figure 2D). All of the mice were sacrificed on day 21 p.i. and the bladder volume and weight were measured. We found that the size and weight of the bladders were significantly decreased in the two drug treatment groups compared to the vehicle control (Figure 2E). We sectioned the bladder tissues of the mice and found that bladder tumor formation in MMC‐ or amiodarone‐treated mice was evidently suppressed compared to the vehicle control by H&E staining (Figure 2F). In the vehicle group, tumor formation as well as cancer cells invasion into the muscle tissue of the bladder were clearly detected (Figure 2F). In addition, we found edema around the mucosa region along with fibrosis of epithelial tissue in the MMC group (the second row, Figure 2F) compared to the amiodarone treatment group. We further conducted plasma biochemical analysis for diverse parameters including blood urea nitrogen (BUN), creatinine, glutamate oxaloacetate transaminase (GOT), and glutamate pyruvate transaminase (GPT). We found that among the four parameters, only GPT was significantly increased in the MMC treatment group compared to the other two groups (Table S1). Furthermore, we evaluated the side effects of intravesical instillation of amiodarone in normal mice without inoculation of cancer cells (Figure S2A). The weight of the mice and their bladders showed no difference in the presence or absence of amiodarone (Figure S2B). Similarly, amiodarone showed negligible differences in plasma biochemical parameters (BUN, creatinine, GOT, and GPT), comparing the treated mice with the untreated mice (Table S2). In summary, both drugs effectively suppressed bladder tumor formation; however, MMC caused more severe side effects in the treated mice, including loss of body weight, edema, and fibrosis of urothelial tissues and increased GPT value compared to the amiodarone‐treated group.

### 3.3 Amiodarone Increases Autophagy Activity in the Bladder Mucosa Region of the Mice and Suppresses Bladder Cell Proliferation

**FIGURE 3:**
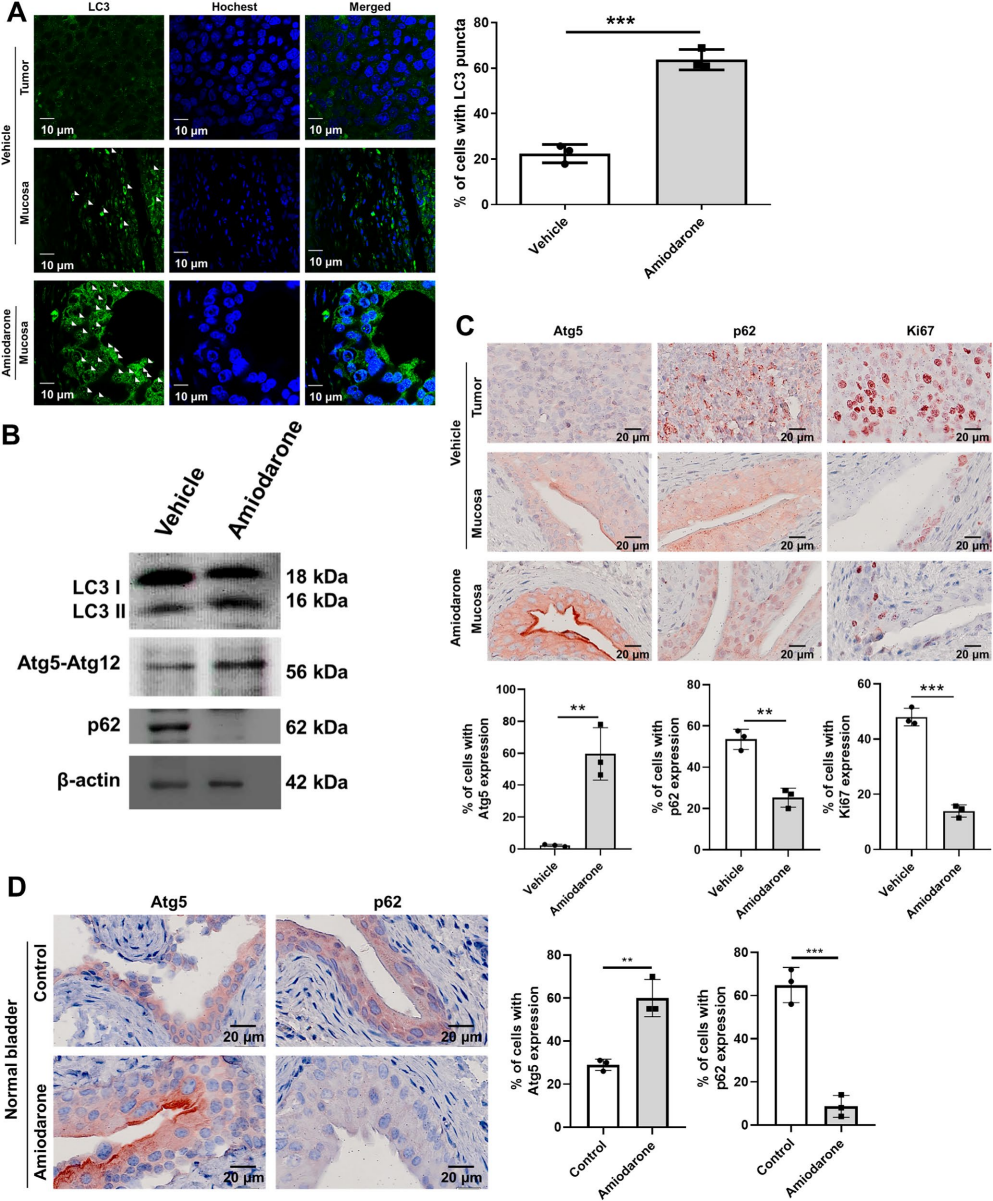
Amiodarone increases autophagy activity in the bladder mucosa region of the mice and suppresses bladder cell proliferation. For normal mice or mice implanted with MB49Luc8 cells, amiodarone (1.5 mg/50 μL) was intravesically instilled into the mice bladders. Mice were sacrificed on day 21 for following analyses. (A) The sections of bladder tissues were stained with anti‐LC3 antibody conjugated with FITC, as shown in green color. The percentage of LC3 puncta in the mucosa tissue related to the total mucosa tissue was quantified. (B) LC3 II, Atg5, and p62 levels were determined by Western blot analysis using specific antibodies. β‐actin was used as the internal control. (C) The sections of bladder tissues were labeled with anti‐Atg5, p62, or Ki67 antibodies followed by IHC assay. (D) The sections of normal bladder tissue were labeled with anti‐Atg5 or p62 antibody followed by IHC staining. Vehicle represents PBS treatment. Control represents PBS treatment of the normal mice. Error bars represent mean ± SD. *p < 0.05; **p < 0.01; ***p < 0.001. Data were analyzed by Student's *t*‐test.

To clarify whether amiodarone suppresses tumor formation through the induction of autophagy activity in vivo, we measured the expression levels of autophagy markers LC3, Atg5, and p62 in the bladder tissues of the mice after various treatments. Our data showed a significantly increased number of LC3 puncta in the mucosa region of the bladder tissues after amiodarone treatment compared to the vehicle bladder tissues (divided into two regions, Mucosa and Tumor) (the first‐column white arrowhead, Figure 3A). We further detected increased LC3 II and Atg5 protein levels and decreased p62 accumulation in the amiodarone‐treated mice bladder tissue compared to the vehicle group by Western blotting (Figure 3B). Similarly, high Atg5 expression and low p62 accumulation in the bladder mucosa region of the amiodarone‐treated mice were detected compared to the vehicle control mucosa region and tumor tissue by IHC staining of the sections (Figure 3C). We further evaluated cell proliferation potential by Ki67 staining (a cell proliferation marker). Our data showed that Ki67 staining of the bladder tumor tissue was the highest; however, the bladder mucosa region in the presence of amiodarone was the lowest compared to the vehicle tumor region (Figure 3C). We also instilled amiodarone into the bladder cavities of the control mice without cancer cell injection as a control. Similarly, we detected increased Atg5 expression and decreased p62 accumulation in the bladder mucosa of the mice (Figure 3D). Our immunofluorescent images and IHC staining imply that low autophagy activity in both tumor and mucosa sections contributed to tumor progression. Additionally, by intravesical instillation treatment, amiodarone directly induced autophagy activity in the mucosa tissue and bladder cancer to suppress mucosa‐derived bladder tumor formation. Altogether, amiodarone was found to induce autophagy activity in the bladder mucosa region of the mice, which correlates with the decrease of the bladder cell proliferation and tumor formation.

### 3.4 Amiodarone‐Induced Autophagy Suppresses Tumor Growth, and Autophagy Deficiency Promotes Bladder Tumor Formation in the Orthotopic Mouse Model

**FIGURE 4:**
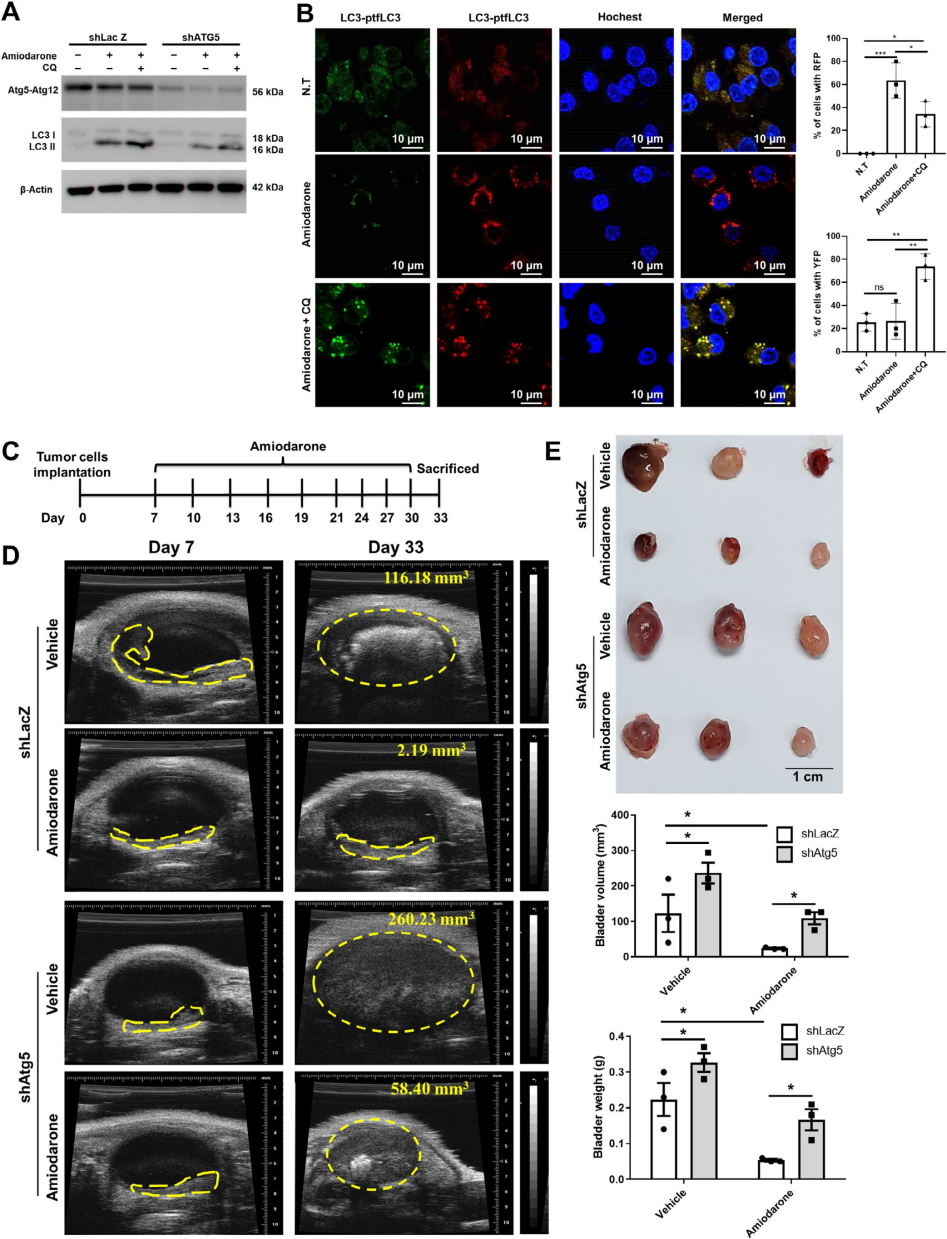
Amiodarone‐induced autophagy suppresses tumor growth, and autophagy deficiency promotes bladder tumor formation in the orthotopic mouse model. (A) MB49Luc cells harboring lentiviral shATG5 or shLacZ gene were established. The levels of LC3II and Atg5 were measured after amiodarone (20 μM) treatment for 24 h followed by CQ (50 μM) treatment for another 24 h by Western blotting using specific antibodies. β‐actin was used as the internal control. (B) MB49Luc cells were transfected with the plasmid DNA (ptfLC3, 4 μg) to investigate autophagic flux. MB49Luc cells harboring ptfLC3 DNA were treated with amiodarone (20 μM) for 24 h followed by CQ (50 μM) blockage for another 24 h and investigated under a fluorescent microscope. Yellow color represents autophagosome formation. Red color represents autolysosomes. Scale bar = 10 μm. (C) The time course of the mice experiment. The stable cells used in (A) were implanted into the mice bladders, and amiodarone (1.5 mg/50 μL) was instilled into the bladders on day 7 p.i. Mice were sacrificed on day 33. (D) Tumor formation in the bladders of the mice was monitored and the tumor volume was measured by MUI system. (E) After mice were sacrificed, the morphology, volume, and weight of the bladders were investigated and quantified. Vehicle represents PBS treatment. Error bars represent mean ± SD. *p < 0.05; **p < 0.01; ***p < 0.001. Data were analyzed by one‐way ANOVA.

To clarify whether amiodarone‐induced autophagy activity regulates bladder tumor formation, we silenced the *Atg5* gene of the MB49Luc cells or control *lac Z* gene using the lentivirus vector and generated MB49LucshAtg5 and MB49LucshLacZ stable cell lines. Our data showed that the protein levels of both Atg5 and LC3 II decreased in the stable MB49LucshAtg5 cells compared to MB49LucshLacZ cells either without or with amiodarone treatment (lane 1 vs. lane 4 and lane 2 vs. lane 5, Figure 4A). We further treated MB49Luc cells with amiodarone for 24 h followed by chloroquine (CQ, a blocker of autophagosome and lysosome fusion) blockage for another 24 h. In Figure 4A, LC3 II accumulation was increased in the presence of CQ compared to the group without CQ (lane 3 vs. lane 2; lane 6 vs. lane 5, Figure 4A), indicating that amiodarone induced a degradative autophagic flux in the bladder cancer cells. We further confirmed that amiodarone induced autophagic flux by performing transient transfection of the plasmid ptfLC3, which contained the LC3 transgene fused with RFP and GFP genes. Under the neutral environment of the autophagosome, both RFP (red) and GFP (green) genes were expressed (yellow). Only the RFP gene was expressed in the autolysosome because the condition was changed to acidic [20]. When the autophagic flux was blocked by CQ, we found the number of yellow puncta dramatically increased (lower panels, Figure 4B). In summary, amiodarone increased autophagy activity and triggered a complete autophagic flux in the bladder cancer cells. We then inoculated the MB49LucshLacZ or MB49LucshAtg5 stable cell line into the mice bladder using the above orthotopic mouse model. Amiodarone or PBS (Vehicle) was intravesically instilled from day 7 p.i. at 3‐day intervals. All the mice were sacrificed on day 33 p.i. (Figure 4C). We found that the image of the tumor nodule of shAtg5 group was larger compared to the shLacZ group without amiodarone treatment under MUI at day 33 p.i. (yellow dash line, 260.23 mm^3^ vs. 116.18 mm^3^, Figure 4D). In the presence of amiodarone, tumor size significantly decreased in either the shLacZ control or in the shAtg5 group (Figure 4D). Notably, the tumor image of the shLacZ control group showed a further decrease in size compared to that of the shAtg5 group in the presence of amiodarone (Figure 4D). Moreover, the shAtg5 group showed higher bladder volume and weight compared to the shLacZ group without amiodarone treatment (row 1 vs. row 3, Figure 4E). In the presence of amiodarone, the volume of the bladders decreased in either the shLacZ control or shAtg5 group (row 2 and row 4, Figure 4E). Notably, the bladder volume of the shLacZ control group was significantly reduced compared to that of shAtg5 group under amiodarone treatment (row 2 vs. row 4, Figure 4E). In summary, our findings support the notion that increased autophagy activity suppresses bladder tumor formation. Kamat and Lamm reported that patients receiving pharmaceutical drugs for bladder cancers experience various side effects [21]. Therefore, we determine the side effects of amiodarone in vivo. Similar to the result in Figure 2D, the body weight of all the mice showed no difference with or without amiodarone treatment (Figure S2C). There were no differences in the plasma biochemical (BUN, creatinine, GOT, and GPT) and hematological parameters (WBC, LY, RBC, Hb, and PLT) between the shAtg5 and shLacZ groups after amiodarone treatment (Table S3). To clarify whether amiodarone instillation into the bladder could be detected in the blood, we measured the levels of amiodarone and desethylamiodarone (the metabolite of amiodarone) in the blood of the mice after treatment. The 8 and 0.16 μg/mL standard analysis showed that desethylamiodarone peaked at 4 min and amiodarone peaked at 10 min using HPLC‐UV. Compared to the peak time of the standard, neither desethylamiodarone nor amiodarone could be detected in the blood of the shAtg5 and shLacZ control groups after either vehicle or amiodarone treatment (Figure S2D). In addition, because amiodarone is an antiarrhythmic drug, we measured the blood pressure of the mice to determine whether it affected mice heart rate. Our data showed that there was no difference between the shAtg5 and shLacZ groups for diastolic pressure, systolic pressure, and pulse on day 33 after amiodarone treatment using a BP‐2000 blood pressure analysis system (Table S4). In conclusion, our results underscore a tumor suppressive role of increased autophagy activity in bladder cancer development and also demonstrate that there was no detectable amiodarone in the blood of these mice and appeared to be no additional influence on the physiological conditions of the mice receiving the intravesical instillation of amiodarone.

### 3.5 Amiodarone‐Induced Autophagy Suppresses the Growth of the Bladder Cancer Cells Through the Caspase‐Dependent Pathway

**FIGURE 5:**
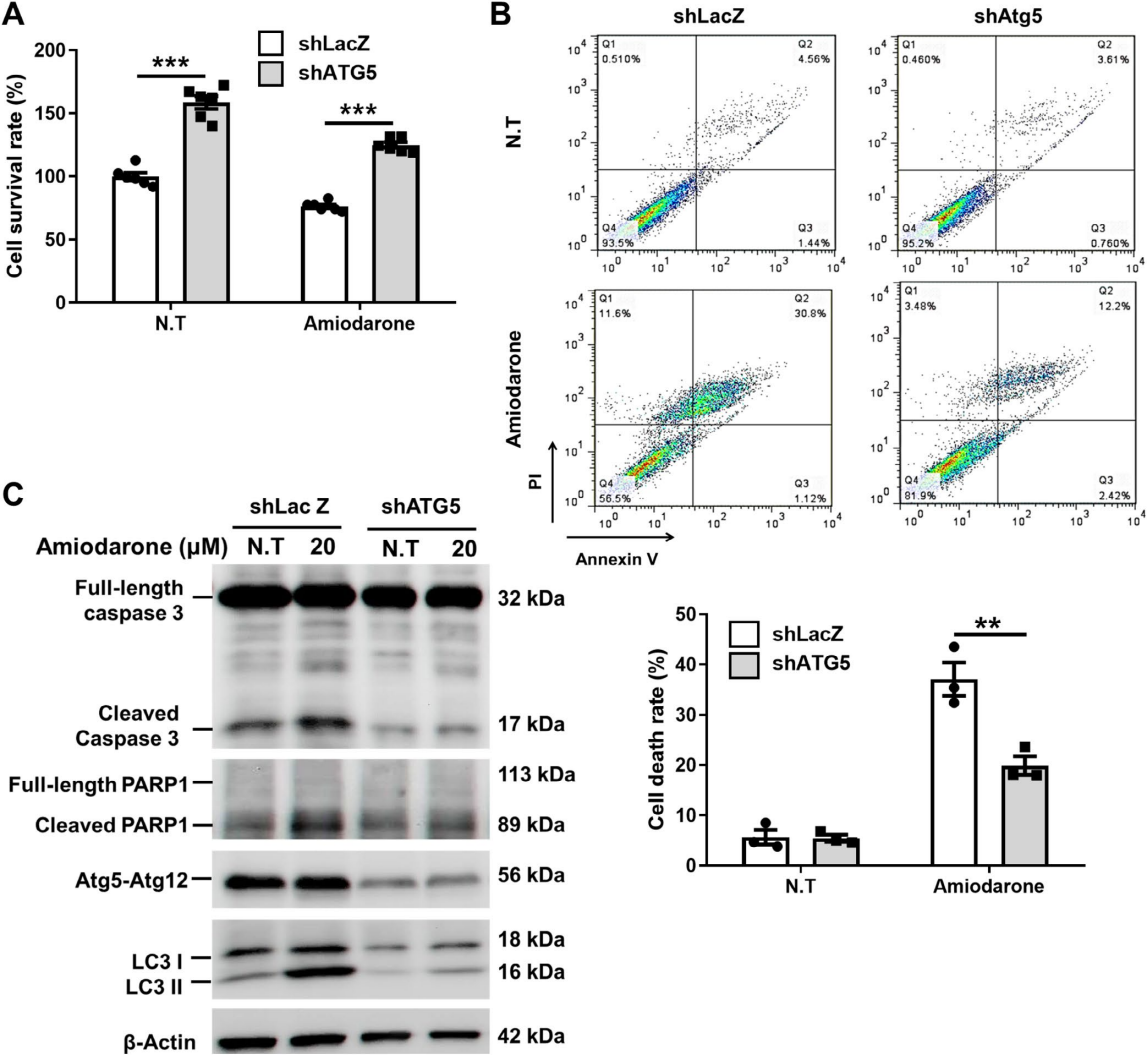
Amiodarone‐induced autophagy suppresses the growth of the bladder cancer cells through the caspase‐dependent pathway. (A, B) The growth and death of shLacZ and shATG5 MB49Luc cell lines were determined by MTT assay (A) and PI‐Annexin V double staining (B) followed by flow cytometry analysis after amiodarone treatment for 24 h. N.T means no treatment. (C) The protein levels of caspase 3, PARP1, Atg5, and LC3 in the MB49Luc stable cell lines after amiodarone treatment for 24 h were evaluated by Western blotting using specific antibodies. β‐actin was used as the internal control. *p < 0.05; **p < 0.01; ***p < 0.001. Data were analyzed by one‐way ANOVA.

Stress‐induced autophagy leads to apoptosis of some cells [22]. We found that the shAtg5 stable cell line showed a higher growth rate compared to the control shLacZ cells without amiodarone treatment, and this suppressive effect was further enhanced in the presence of amiodarone (Figure 5A). This finding is consistent with the result of Figure 4E. Altogether, it indicates that the autophagy‐related *Atg5* gene and induction of autophagy activity play a suppressive role in cell proliferation. To clarify whether the two stable cell lines died of apoptosis in the presence of amiodarone, our flow cytometry analysis revealed that increased autophagy by amiodarone causes more apoptotic cell death in both shLacZ and shAtg5 cell lines compared to their individual cells without amiodarone (30.8% vs. 4.56% and 12.2% vs. 3.61%, Figure 5B). Moreover, shAtg5 cells showed a lesser amount of apoptotic cell population compared to shLacZ cells with or without amiodarone (3.61% vs. 4.56% and 12.2% vs. 30.8%, Figure 5B), indicating that the *Atg5* gene suppresses cell proliferation and no evident apoptotic cell death was detected in these two cell lines in the absence of amiodarone (4.56% vs. 3.61%, Figure 5B).

We further showed that the levels of cleaved caspase 3, cleaved PARP1, and LC3 II protein increased in the presence of amiodarone for 24 h in shLacZ cells (lane 2 vs. lane 1, Figure 5C). However, these increases were dramatically reduced in shAtg5 cells (lane 3 vs. lane 4, Figure 5C). In summary, our results imply that the caspase 3‐dependent pathway is involved in amiodarone‐related death of bladder cancer cells.

## 4 Discussion

**FIGURE 6:**
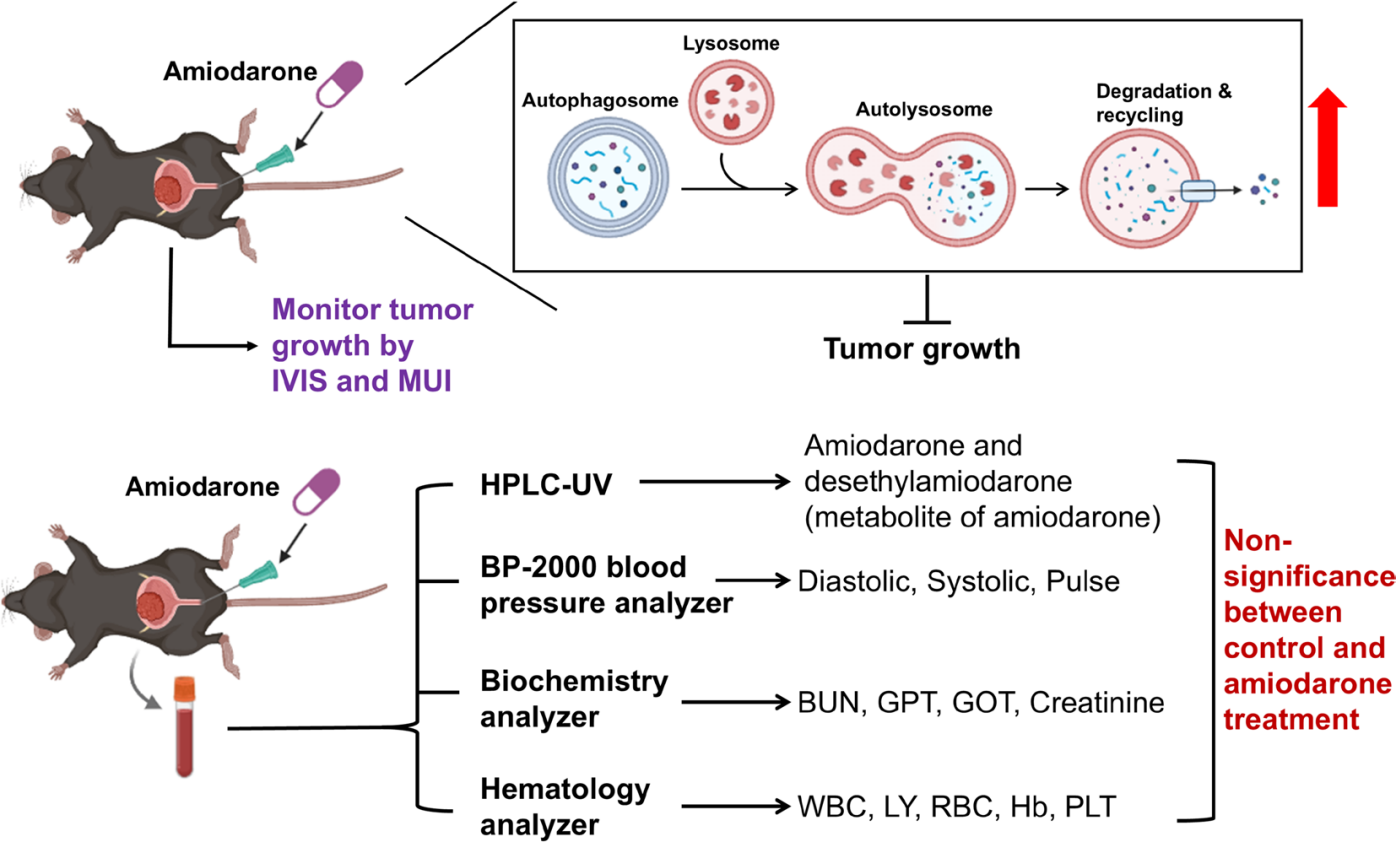
Schematic model illustrating the tumor‐inhibitory effects of amiodarone by activating autophagic activity and amiodarone delivered by intravesical route showing negligible influence on the physiologic conditions of the treated mice. Amiodarone administered intravesically into the urinary bladder of mice induces autophagic activity, which suppresses bladder tumor formation. This effect is monitored noninvasively using IVIS and MUI (upper). Plasma biochemistry parameters, hemograms, blood pressure, and blood concentrations of amiodarone and desethylamiodarone were analyzed in treated mice. Amiodarone administered via the intravesical route demonstrated a negligible impact on the physiological conditions of the treated mice (lower).

We utilized specimens of bladder cancer patients and an orthotopic murine model as well as mouse bladder cancer cell lines to demonstrate increased autophagy suppressing bladder tumorigenesis (Figure 6). Moreover, we confirmed the sensitivity and reliability of IVIS and MUI are equivalent in monitoring bladder tumor formation in a spatiotemporal manner in mice. Furthermore, repurposing amiodarone as an autophagy inducer enhanced autophagy activities both in vivo and in vitro.

Autophagy plays a dual role during tumor development of diverse cancers. In the early phase of tumor formation, autophagy suppresses tumorigenesis by limiting ROS production, DNA damage, and proliferation [22]. However, in the late stage of tumor progression, autophagy promotes tumorigenesis by activating the metabolic pathways to overcome the stress conditions. Due to the lack of optimal animal models and reliable noninvasive monitoring tools to investigate the tumor development in a temporal and spatial manner, it remains contradictory regarding the effect of autophagy on bladder tumorigenesis [22].

The syngeneic orthotopic mouse model of bladder tumor formation is valuable for monitoring bladder tumor formation and evaluating the efficacy of anti‐cancer drugs because it mimics real bladder tumor formation in humans. Noninvasively monitoring image systems allow spatiotemporal investigation of tumor formation in vivo. In addition to mouse bladder cancer MB49 cells expressing firefly luciferase gene used in this study, we further established another MB49 cell line expressing the NanoLuc luciferase gene (deep sea shrimp, Promega), which produces glow‐type luminescence that is > 100‐fold brighter than the firefly luciferase gene used here. Further study is ongoing to confirm whether it can shorten the detection time of early tumor formation.

Intravesical drug instillation is widely used in clinical bladder cancer treatment to suppress tumor recurrence as well as blocking tumor progression in the early stage. To mimic the clinical treatment of bladder cancer patients, we also used the drug instillation protocol to treat the bladder tumors in the orthotopic mouse model. Our data demonstrated that wild type bladder cancer MB49 cells‐induced tumor formation was effectively suppressed by amiodarone. Furthermore, the suppressive effect on tumor formation mediated by increased autophagy activity was reversed by silencing the *Atg5* gene (Figure 4D,E), indicating that both autophagy and the *Atg5* gene itself play a suppressive role in bladder cancer development. Moreover, we found that *Atg5* also plays a dual role in tumorigenesis. It suppressed tumor formation and cell migration under normal autophagy conditions. In contrast, *Atg5* plays a pro‐tumor role under autophagy deficiency conditions [23]. We also found that low autophagy activity was correlated with increased tumor grade and poor overall survival of bladder cancer patients (Figure 1). Similar findings have been reported in various types of tumors [22]. Altogether, our findings sustain the notion that autophagy plays an anti‐tumor role in the early stage of bladder tumor formation.

We previously reported that amiodarone as a repurposing drug is a potent autophagy inducer and can effectively suppress liver tumor formation through selective degradation of oncogenic factors *mir‐224* and cyclin D1 [9, 10]. Similarly, cyclin D1 has been reported to be highly expressed in Ta/T1 bladder cancer [24] and we found low autophagy activity in bladder cancer specimens. Taken together, above findings suggest that autophagy degradation machinery may suppress bladder tumor formation through the degradation of cyclin D1, which warrants further exploration. This study also revealed that autophagy‐induced caspase 3 cleavage and apoptosis are involved in the inhibition of cell proliferation after amiodarone treatment. However, the possible mechanism by which amiodarone induces autophagy and apoptosis in bladder cancer remains elusive. Pyo et al. reported that Atg5 inhibits the extrinsic apoptosis pathway through interrupting the interaction between FADD and DISC, indicating that Atg5 plays a crucial role in IFN‐gamma‐induced autophagic cell death [25]. On the other hand, Yousefi et al. reported that calpain‐mediated Atg5 cleavage switches autophagy to apoptosis, which occurred independent of cell type. Truncated Atg5 translocated from the cytosol to the mitochondria, where it associated with the anti‐apoptotic molecule Bcl‐xL. This association subsequently triggered cytochrome c release and caspase activation without the activation of autophagy [26]. Consistent with this finding, Trichokonin VI, an antimicrobial peptide, triggers the influx of extracellular calcium, thereby facilitating calpain‐dependent apoptosis in liver cancer cells [27]. Moreover, administration of Trichokonin VI TK induces reactive oxygen species accumulation to result in the subsequent disposal of damaged mitochondria within autophagosomes through Atg5‐mediated and mitochondria‐selective autophagy [27]. A recent study showed that high concentration of 20‐hydroxyecdysone elevates the concentration of calcium to induce cleavage of ATG5, thereby stimulating the transition from autophagy to apoptosis [28]. In summary, these findings demonstrated that calpain‐mediated Atg5 cleavage contributes to the transition from autophagy to apoptosis, suggesting a molecular link between autophagy and apoptosis. In fact, amiodarone can increase the concentration of intracellular calcium [29], implying that amiodarone may promote calcium‐dependent calpain activation to mediate Atg5 cleavage, thereby resulting in the transition from autophagy to apoptosis.

In this study, we intend to identify repurposing drugs which could block early stage bladder tumor formation through manipulating autophagy activity and have minor influence on the normal physiological conditions. Currently, immunotherapy by intravesical BCG causes localized inflammation, which leads to chemical cystitis and macroscopic hematuria [30]. The pharmaceutical drugs for bladder cancer such as MMC have diverse side effects, including contracted bladder, bladder wall calcification, contact dermatitis, chemical cystitis, hematuria, diarrhea, and neuropathy [30].

Amiodarone is widely used as an antiarrhythmic drug with adverse effects, such as acute and chronic pulmonary damage [31]. From an immunological perspective, amiodarone may cause an imbalance between Th1 and Th2 subpopulations and increase the levels of tumor necrosis factor alpha (TNF‐α) and transforming growth factor beta (TGF‐β) [32]. Additionally, amiodarone seems to induce thrombocytopenia, which may lead to aplastic anemia [33] due to the long half‐life of amiodarone. Amiodarone has a long and variable terminal half‐life, approximately 9 to 77 days, due to its high lipophilicity and slow rate of release from adipocytes, indicating that the side effects may persist for months after amiodarone is discontinued [34]. Therefore, it is important to clarify the possible side effects of amiodarone in mice with or without bladder tumor in this study. Compared to the clinical amiodarone treatment via oral or intravenous route, the intravesical instillation route showed minimal side effects on the normal physiological parameters of mice. Amiodarone is metabolized to desethylamiodarone, which easily penetrates tissues barriers including the lungs [35]. As shown in Figure S2D, both amiodarone and its metabolite desethylamiodarone in the blood of the mice were undetectable by HPLC‐UV analysis, indicating that intravesical instillation of amiodarone into the bladder did not result in systemic absorption and its efficacy is limited to the superficial area of the bladder. Furthermore, because amiodarone is originally an antiarrhythmic drug, we measured the blood pressure of the mice, and found no difference in diastolic pressure, systolic pressure, or pulse on day 33 after amiodarone treatment, based on readings obtained using a BP‐2000 blood pressure analysis system (Table S4). Our findings are consistent with results reported by Boulin et al. and Guiu et al. patients with hepatocellular carcinoma were safely treated with 150 mg of amiodarone via the intravenous route [36, 37]. Moreover, according to the database from National Health Research Institutes in Taiwan, liver cancer patients with a history of amiodarone exhibited the expanded survival time compared to the group of non‐amiodarone users [38]. However, the dosage of amiodarone to induce autophagy for suppressing bladder tumor formation through intravesical instillation still needs to be examined and determined. The standard‐of‐care treatment for bladder cancer typically includes mitomycin C, cisplatin or gemcitabine‐based chemotherapy, followed by radical cystectomy. Current chemotherapeutic agents focus on cytotoxic drugs; thus, autophagy‐based therapy can provide an alternative option. Preclinical studies and our findings suggest that amiodarone‐induced autophagy and low side effects offer a promising strategy for combination therapy to improve patient outcomes.

## Funding

This study was supported by grants from the National Science and Technology Council, Taipei, Taiwan (NSTC 112‐2320‐B‐A49‐025‐MY3); the Cancer and Immunology Research Center of National Yang Ming Chiao Tung University from The Featured Areas Research Center Program within the framework of the Higher Education Sprout Project by the Ministry of Education (MOE), Taiwan (112W31101; 113W031101; 112W10161).

## Conflicts of Interest

The authors declare no conflicts of interest.

## Supporting information


**Data S1:** Supporting Information.

## Data Availability

The data that support the findings of this study are available on request from the corresponding author. The data are not publicly available due to privacy or ethical restrictions.
