# Supplementary material for: Autophagy Plays a Suppressive Role in Bladder Tumor Formation in an Orthotopic Mouse Model and Bladder Cancer Patient Specimens
Source: Kaohsiung J Med Sci. 2026 Jan 23;42(8):e70179. doi: 10.1002/kjm2.70179 (PMC13399893; doi:10.1002/kjm2.70179)
Supplement: Supplementary file 1 [file KJM2-42-e70179-s001.docx]

**Autophagy plays a suppressive role in bladder tumor formation in an orthotopic mouse model and bladder cancer patient specimens**

Wan-Ting Kuo, Chin-Chen Pan, Yi-Wen Liu, Nan-Haw Chow, Hong-Lin Cheng, Shan-Ying Wu, Sheng-Hui Lan, Chih-Peng Chang, and Hsiao-Sheng Liu

**Supplemental Data**

**Supplementary Figure S1.**

**Supplementary Figure S2**

**Supplementary Figure S3**

**Supplementary Table S1**

**Supplementary Table S2**

**Supplementary Table S3**

**Supplementary Table S4**

**Supplementary Table S5**

**Supplementary Figure S1. Characterization of stable bladder cancer cells expressing luciferase gene and assessment of bladder tumor formation in mice by IVIS, MUI system, and H&E staining in the orthotopic mouse bladder tumor model.**

(A) The luciferase activity of four stable clones of MB49Luc cells was determined. The cells (1x10^4^) were seeded in a 24-well tray and cell lysates were (3 μg) measured with a luminometer. (B) Quantification of bioluminescence of four MB49-Luc stable clones of three different cell numbers in a 96-well plate under IVIS. (C) Colony formation of two MB49-Luc stable clones (2x10^4^) in a 6-well plate. The colony number was counted under a light microscope on day 14. (D) MB49Luc8 stable cells (1x10^7^) were inoculated into the mice bladders. The bioluminescence of the inoculated mice was assessed on days 7 and 14 p.i. under IVIS. (E) MB49Luc8 cells (1x10^7^) were implanted into the bladders of the mice and monitored under the IVIS and MUI system. The mice were sacrificed on days 4, 13, and 21, and bladder tissues were sectioned and stained by H&E staining. T: tumor. N: non-tumor. N.T. represents no MB49Luc8 cell inoculation. Error bars represent mean ± SD. *p < 0.05; **p < 0.01; ***p < 0.001. Data were analyzed by one-way ANOVA.

**Supplementary Figure S2. Amiodarone showed negligible effects on the physiological parameters of the mice.**

(A) The time course of mice experiment. Mice received amiodarone (1.5 mg/50 μl) by intravesical instillation on day 4 p.i. and at 3-day intervals until day 19 p.i. Mice were sacrificed on day 21 p.i. Control represents PBS treatment of the mice. (B) The morphology, weight of the bladders, and the body weight of the mice were measured. (C) The body weight of the mice after intravesical amiodarone instillation was measured for 21 days. (D) After mice were sacrificed on day 33 p.i., the plasma of mice was analyzed by HPLC analysis. Blank plasma represents acetonitrile and plasma. Amiodarone and desethylamiodarone at the concentrations of 8 μg/ml and 0.16 μg/ml were used as the standards. Vehicle represents PBS treatment. Error bars represent mean ± SD. *: *p < 0.05; **p < 0.01; ***p < 0.001. Data were analyzed by Student’s t-test.

**Figure S3. The association between autophagy activity and cancer progression.**

**(A-B)** 57 out of 289 patients were analyzed for observing the protein levels of Atg5 and p62 in tumor grades and tumor stages. All results were measured by IHC staining and were quantified by HistoQuest analysis software. The percentage of Atg5 and p62 expression in bladder tissues was calculated by cells with protein expression vs. all nucleated cells.

**Supplementary Table S1. Plasma biochemical parameters of the mice at the end of the treatment.**

C57BL/6 mice were inoculated with the stable MB49Luc cell lines. Amiodarone or MMC was intravesically instilled into the bladders of the mice on day 4 p.i. Values are mean ± SD. *: p < 0.05.

|  | Vehicle | Mitomycin C | Amiodarone |
| --- | --- | --- | --- |
| Blood urea nitrogen (BUN)(mg/dL)  Creatinine (mg/dL)  Glutamate oxaloacetate transaminase (GOT) (U/L)  Glutamate pyruvatetransaminase (GPT) (U/L) | 26.5±5.8   0.2±0.0   82±1.0   21.3±5.1 | 15.9±1.1   0.15±0.1   66±18.5  **41.3±8.1*** | 17.0±1.2   0.18±0.1   78±4.4   22.3±1.2 |

**Supplementary Table S2. The plasma biochemical parameters of the mice without tumor at the end of the treatment.**

C57BL/6 mice without implantation of bladder cancer cell lines were intravesically instilled with amiodarone (1.5 mg/50 μl) or PBS. Controls were treated with PBS. Values are mean ± SD. *: p < 0.05.

|  | Mice without inoculation of cancer cells | |
| --- | --- | --- |
|  | Control | Amiodarone |
| Blood urea nitrogen (BUN)(mg/dL)  Creatinine (mg/dL)  Glutamate oxaloacetate  transaminase (GOT) (U/L)  Glutamate pyruvate  transaminase (GPT) (U/L) | 19.1 ± 3.4    0.2 ± 0.05    118.3 ± 33.6    39.6 ± 11.6 | 17.9 ± 0.8    0.2 ± 0    70.6 ± 0.5    22.6 ± 2.5 |

**Supplementary Table S3. Plasma biochemical parameters and hemograms of the mice at the end of the treatment.**

C57BL/6 mice were inoculated with the stable MB49Luc cell lines harboring lentiviral shLacZ or shAtg5 gene in the bladders. WBC: white blood cell; Ly: lymphocyte; RBC: red blood cell; Hb: hemoglobin concentration; PLT: platelets; Values are mean ± SD. *: p < 0.05.

|  | shLacZ | | shAtg5 | |
| --- | --- | --- | --- | --- |
|  | Vehicle | Amiodarone | Vehicle | Amiodarone |
| **Biochemical parameters**  Blood urea nitrogen (BUN)(mg/dL)  Creatinine (mg/dL)  Glutamate oxaloacetate  transaminase (GOT) (U/L)  Glutamate pyruvate  transaminase (GPT) (U/L)    **Hemograms**  WBC (K/μL)  LY(K/μL)  RBC(M/μL)  Hb(g/dL)  PLT(K/μL) | 40.3 ± 21.9  0.3 ± 0.06    149.3 ± 66.5    37.7 ± 2.3      3.8 ± 0.0  2.5 ± 0.4  9.9 ± 0.1  13.4 ± 0.5  798.3 ± 184.3 | 40.9 ± 18.2  0.4 ± 0.1    117.0 ± 72.0    33.3 ± 17.9      3.8 ± 1.4  1.9 ± 0.2  10.1 ± 1.4  13.4 ± 1.5  785.7 ±158.1 | 35.2 ± 3.9  0.2 ± 0.06    123.0 ± 52.1    58.3 ± 15.5      2.8 ± 1.2  1.5 ± 0.3  9.4 ± 0.2  12.3 ± 0.3  696.3 ± 15.0 | 27.8 ± 3.8   0.3 ± 0.15    103.0 ± 62.9    40.7 ± 18.7      2.6 ± 0.5  1.5 ± 0.1  9.3 ± 0.3  12.3 ± 0.5  686.0 ± 54.8 |

**Supplementary Table S4. The diastolic pressure, systolic pressure, and pulse of the mice at the end of the treatment.**

C57BL/6 mice were inoculated with the stable MB49Luc cell lines harboring lentiviral shLacZ or shAtg5 gene in the bladders. Values are mean ± SD. *: p < 0.05.

|  | shLacZ | | shAtg5 | |
| --- | --- | --- | --- | --- |
|  | Vehicle | Amiodarone | Vehicle | Amiodarone |
| Diastolic  (mmHg)  Systolic  (mmHg)  Pulse  (Heartbeats per minute) | 67 ± 13.8    113.7 ± 2.8    628.7 ± 28.6 | 64.6 ± 17.0    112.7 ± 6.2    628.3 ± 42.7 | 64.3 ± 10.1    107.2 ± 6.6    653.8 ± 58.2 | 67.4 ± 12.6    105.1 ± 5.8    644.7 ± 36.8 |

**Supplementary Table S5. The volume of tumor in the bladders of mice was determined by MUI image system.**

No. 7 mouse died of bladder tumor progression. 0 means no tumor was detected.

|  | Tumor volume (mm^2^) | | |
| --- | --- | --- | --- |
| No. | N.T | MMC | Amiodarone |
| 1 | 38.12 | 2.95 | 1.15 |
| 2 | 41.59 | 2.71 | 1.16 |
| 3 | 2.17 | 1.39 | 1.76 |
| 4 | 28.96 | 1.43 | 1.32 |
| 5 | 1.9 | 2.4 | 0.93 |
| 6 | 0 | 1.94 | 1.39 |
| 7 | Death | 3.23 | 2.06 |
